# Effect of Pregabalin and Dexamethasone on Postoperative Analgesia after Septoplasty

**DOI:** 10.1155/2014/850794

**Published:** 2014-04-24

**Authors:** Abdullah Demirhan, Akcan Akkaya, Umit Yasar Tekelioglu, Tayfun Apuhan, Murat Bilgi, Veysel Yurttas, Hakan Bayir, Isa Yildiz, Uzeyir Gok, Hasan Kocoglu

**Affiliations:** ^1^Department of Anesthesiology and Reanimation, Faculty of Medicine, Abant Izzet Baysal University, Golkoy, 14280 Bolu, Turkey; ^2^Department of Otorhinolaryngology, Faculty of Medicine, Abant Izzet Baysal University, Golkoy, 14280 Bolu, Turkey

## Abstract

*Objectives*. The aim of this study was to explore effect of a combination of pregabalin and dexamethasone on pain control after septoplasty operations. *Methods*. In this study, 90 patients who were scheduled for septoplasty under general anesthesia were randomly assigned into groups that received either placebo (Group C), pregabalin (Group P), or pregabalin and dexamethasone (Group PD). Preoperatively, patients received either pregabalin 300 mg one hour before surgery, dexamethasone 8 mg intravenously during induction, or placebo according to their allocation. Postoperative pain treatment included tramadol and diclofenac sodium 30 minutes before the end of the operation. Numeric rating scale (NRS) for pain assessment, side effects, and consumption of tramadol, pethidine, and ondansetron were recorded. *Results*. The median NRS score at the postoperative 0 and the 2nd h was significantly higher in Group C than in Group P and Group PD (*P* ≤ 0.004 for both). The 24 h tramadol and pethidine, consumptions were significantly reduced in Groups P and PD compared to Group C (*P* < 0.001 and *P* < 0.001). The incidence of blurred vision was significantly higher in Group PD compared to Group C within both 0–2 h and 0–24 h periods (*P* = 0.002 and *P* < 0.001, resp.). *Conclusions*. We conclude that administration of 300 mg pregabalin preoperatively may be an adequate choice for pain control after septoplasty. Addition of dexamethasone does not significantly reduce pain in these patients.

## 1. Introduction

Postoperative pain remains a major problem after septoplasty operations despite improvements in algological and surgical techniques. Appropriate management of postoperative pain is known to reduce the length of the hospital stay and to make patients more comfortable by reducing pain-associated complications [1]. Multimodal analgesia is a common method used to improve the analgesic efficacy and to reduce the dosage and side effects of drugs [1].

Pregabalin acts as a synthetic analog of the neurotransmitter gamma-aminobutyric acid (GABA) with analgesic, anticonvulsant, and anxiolytic effects [2]. Its antihyperalgesic efficacy has been shown in a human pain model [3]. The oral bioavailability of pregabalin is approximately 90%. After oral administration of the drug, maximum plasma concentration can be achieved within 1 h [2]. Pregabalin has been found to be effective in controlling postoperative pain and in decreasing analgesic consumption [4, 5]. Glucocorticoids have strong anti-inflammatory effects and exhibit antiemetic and analgesic efficacy [6]. Dexamethasone was reported to increase the efficacy of analgesia when given alone or in combination with other drugs [7, 8]. The aim of this study was to determine the efficiency of pregabalin alone and the combination with dexamethasone to the septoplasty operations regarding postoperative pain control and analgesic consumption.

## 2. Materials and Methods

This prospective study was approved by the Institutional Ethics Committee and conducted in accordance with the ethical principles described by the Declaration of Helsinki. After obtaining written informed consent, 90 ASA I-II physical status patients aged 18–75 years who were awaiting elective septoplasty were included in this prospective, randomised, and double-blind study. The exclusion criteria were patients who failed to cooperate, regularly used certain drugs (corticosteroids, benzodiazepines, tricyclic antidepressants, NSAIDs, or other analgesic drugs), and had a history of allergy to any of the study medications, peptic ulcer disease or diabetes mellitus, or significant cardiac, pulmonary, hepatic, or renal disease. Pregnant women and any patient with a body mass index >35 kg/m^2^ were also excluded from the study.

Preoperative visits were performed for all the patients, and the patients were instructed in the use of a numeric rating scale (NRS) for pain assessment (0 cm: no pain; 10 cm: worst pain imaginable) and patient-controlled analgesia (PCA).

The patients were randomly divided into one of the three groups (30 patients each) using a computer-based randomisation system: Group P (pregabalin group), Group PD (pregabalin plus dexamethasone group), and Group C (control group). The computer-generated randomisation list was delivered to a nurse who was unaware of the study. Pregabalin (Lyrica 300 mg capsule, Pfizer, Istanbul, Turkey) was given orally with 10 mL of water 1 h before surgery to the patients in Group P and Group PD, and a similar looking placebo capsule was given to the patients in Group C. The exact same packaging was used for the placebo capsules, as the active capsules.

On arrival in the operating room, intravenous (i.v.) access was established with a 20 G i.v. cannula, and the ECG, blood pressure, and peripheral oxygen saturation (SpO_2_) were monitored. Induction of anaesthesia was started with a 1 min infusion of remifentanil (Ultiva 1 mg mL^−1^, GlaxoSmithKline, Istanbul, Turkey) at a dose of 1 mcg kg^−1^ min. Afterwards, the infusion dose of remifentanil was reduced to 0.5 mcg kg min^−1^. The induction of anaesthesia was achieved with propofol (Propofol Lipuro %1, B. Braun Irengun, Istanbul, Turkey) 2 mg kg^−1^, and rocuronium (Myocron, Vem, Istanbul, Turkey) 0.6 mg kg^−1^ was used for muscle relaxation. After endotracheal intubation and stabilisation, 2 mL dexamethasone (Deksamet 2 mL, 4 mg mL^−1^, Osel, Istanbul, Turkey) was administered intravenously to the Group PD patients. Other study groups received 2 mL i.v. saline solution as a placebo. Sevoflurane was used for maintenance of anesthesia. The patients were mechanically ventilated to maintain their end-tidal CO_2_ values between 34 and 38 mmHg. The infusion of remifentanil was adjusted to keep the mean arterial pressure (MAP) at 65–100 mmHg. The fluid infusion was increased when the MAP fell below 60 mmHg. If hypotension occurred, the infusion of remifentanil was stopped, and ephedrine HCl i.v. 10 mg (Ephedrine 0.05 mg mL^−1^, Osel, Istanbul, Turkey) was given. In the event of bradycardia, i.v. atropine sulfate (Atropine. 1 mg mL^−1^, Galen, Istanbul, Turkey) 0.5 mg was administered.

The operations were performed by two surgeons. The surgeons injected a mixture of lidocaine HCl (20 mg mL^−1^) and epinephrine (0.0125 mg mL^−1^) locally to the septal tissue before the surgery. Half an hour before the end of the surgery, 50 mg of tramadol HCl was given i.v. for the treatment of postoperative pain. At the same time, 75 mg of diclofenac sodium (Diclomec, 75 mg mL^−1^, Abdi Ibrahim, Istanbul, Turkey) was injected intramuscularly. When the operation was completed, the remifentanil infusion and the sevoflurane inhalation were discontinued and the patient was ventilated with 100% O_2_ at a fresh gas flow rate of 5 L min^−1^. Residual neuromuscular block was reversed with a combination of atropine sulphate (0.015 mg kg^−1^) and neostigmine (40 mcg kg^−1^). The patients were extubated in the operation room when adequate spontaneous ventilation was established. The total remifentanil dose, duration of surgery, and haemodynamic data were recorded. Thereafter, the patients were admitted to the postanaesthetic care unit for at least 1 h until complete recovery. After arrival in the postanesthesia care unit, the patients were connected to a PCA device (Abbot Pain Management Provider, Abbot Laboratories, North Chicago, IL, USA) and administered with tramadol HCl (20 mg bolus dose, 45 min lockout time) via this device. Assessment of pain scores, emetic symptoms, and side effects was made by one of the authors blinded to the patient group. Pethidine HCl (Aldolan 100 mg 2 mL^−1^, Gerot) was administered 0.5 mg kg^−1^ intravenously as a rescue analgesia if the NRS value was >4. Nausea and vomiting were treated with i.v. ondansetron HCl (Zofran 4 mg 2 mL^−1^, GlaxoSmithKline, Istanbul, Turkey).

Intraoperative heart rate and mean arterial blood pressure values were recorded at preoperative, postinduction, and postintubation periods and every 5 minutes intraoperatively. The NRS values were recorded at the 30th min and the 1st, 2nd, 4th, 8th, 12th, and 24th h postoperatively. The amount of pethidine used, the number of patients who received pethidine, the time of the first administration of pethidine, side effects (nausea, vomiting, dizziness, blurred vision, headache, loss of concentration, itching, and others), tramadol consumption between the 0-1st, 1–12th, and 12–24th h, and total tramadol consumption were recorded by another author blinded to the patient group. PCA was ended on the second day, and diclofenac was started, given orally twice a day for analgesia.

## 3. Statistical Methods and Sample Size

The data analysis was performed with SPSS statistical software (SPSS Inc., Chicago, IL, USA) version 11.5 for Windows. The Kolmogorov-Smirnov test was used to determine the normal distribution of the continuous variables, and Levene's test was used to test for the violation of the assumption of homogeneity of variance. The data are shown as the mean ± standard deviation for the continuous variables, the median (minimum–maximum) for the ordinal ones, and the frequency with percent for the categorical ones. The means were compared using a one-way analysis of variance (ANOVA) where appropriate. The median values were compared with the Kruskal-Wallis test. When the *P* values from the ANOVA and the Kruskal-Wallis tests were statistically significant, Tukey's HSD post hoc test or Conover's nonparametric multiple comparison test was used to determine which measurements differed from the others. Where appropriate, categorical comparisons were made using the chi-square, Fisher's exact test, or the likelihood ratio test. A *P* value less than 0.05 was considered statistically significant. The Bonferroni adjustment was used for all multiple comparisons to control type I errors.

### 3.1. Sample Size Estimation

The primary endpoint of this 11 study was to compare tramadol consumption by means of differences in 24-hour among groups. According to the pilot study, in a one-way ANOVA study, sample sizes of 29, 29, and 29 are obtained from the 3 groups whose means are to be compared. The total sample of 87 subjects achieves 90.9% power to detect differences among the means versus the alternative of equal means using an *F*-test with a 0.05 significance level. The size of the variation in the means is represented by their standard deviation which is 21.31. The common standard deviation within a group is assumed to be 54.07. Sample size estimation was performed by using NCSS and PASS 2000 (Kaysville, UT, USA) software.

## 4. Results

Ninety patients were included in the study. The patients were randomly assigned to three groups. There were no statistically significant differences between the groups in terms of their age, sex, demographics, and clinical characteristics (*P* > 0.05 for all), as shown in Table 1.

There was no statistically significant difference between the groups in terms of mean heart rate and mean arterial blood pressure at all-time points for all groups (*P* > 0.00625).

**Figure 1:**
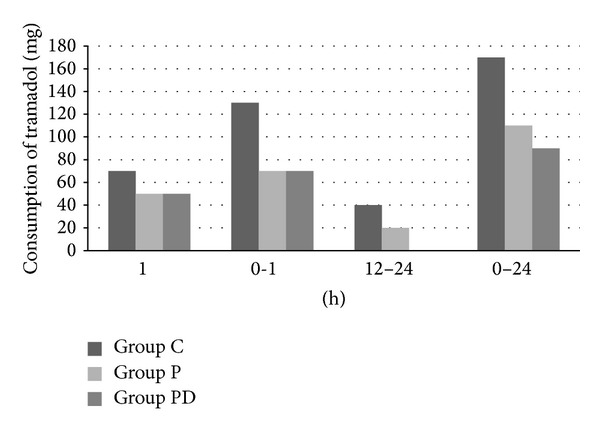
Consumption of tramadol.

Group P and Group PD showed statistically significant lower tramadol consumption compared to Group C within the postoperative 0–24 h period (*P* < 0.001 for all). There was no statistically significant difference between the Group P and Group PD in terms of tramadol consumption (*P* > 0.05 for all).  Table 2 shows the mean tramadol consumption (mg) of the groups within the first 24 h period (Figure 1).

There was no statistically significant difference between the groups in terms of median NRS scores except at the 0 and the 2nd h. The median NRS score at the 0 and the 2nd h was significantly higher in Group C than in Group P and Group PD (*P* ≤ 0.004 for both).  Table 3 shows the mean NRS scores within the first postoperative 24 h.

The requirement ratio of pethidine use in Group P and Group PD was significantly lower than in Group C (*P* < 0.001 for both). The time when pethidine had been administered was not different among the groups (*P* = 0.767). Ondansetron use ratio was significantly lower in patients in Group P compared to Group C (*P* = 0.017). The time when ondansetron had been administered in patients who were given ondansetron was not significantly different among the groups (*P* = 0.410).  Table 4 shows the frequency of postoperative rescue analgesia and ondansetron use among the groups.

The frequency of nausea was higher in Group C and Group PD compared to Group P between the postoperative 0–2 h periods (*P* = 0.007 and *P* = 0.015, resp.). The patients in Group C showed a significantly higher incidence of nausea compared to Group P within the postoperative 0–24 h period (*P* = 0.017). The incidence of blurred vision was significantly higher in Group PD compared to Group C within both 0–2 h and 0–24 h periods (*P* = 0.002 and *P* < 0.001, resp.). There were no statistically significant differences among the groups in terms of other complications (*P* > 0.05 for all).  Table 5 highlights the incidence of observed adverse effects in the groups.

## 5. Discussion

This prospective, randomised, and double-blind study showed that preoperative administration of pregabalin or pregabalin plus dexamethasone improved early pain control and decreased tramadol consumption within the postoperative period after septoplasty. Moreover, pregabalin or pregabalin plus dexamethasone reduced the frequency of rescue analgesia.

Septoplasty is a commonly performed surgical procedure, and patients who undergo septoplasty often experience early postoperative pain [9, 10]. Nasal tampons may add more pain in addition to surgery-induced pain [11]. The efficacy of opioids alone or in combination with NSAIDs in the management of postoperative pain is well known. The multimodal analgesic regimen provides efficient analgesia and reduces opioid usage and the incidence of side effects [12]. Tramadol, an atypical opioid, is widely used in the treatment of moderate-to-severe postoperative pain [13]. Tramadol may cause less respiratory depression and less addictive properties than the other opioids [13]. This drug has become very popular in postoperative pain control due to its safe side effect profile. In common with pregabalin and dexamethasone, antinociceptive effects of tramadol have been reported [14, 15]. The combined use of these drugs is more effective than the use of each agent alone in pain management [14, 15].

Sener et al. [16] investigated the efficacy of lornoxicam, diclofenac, ketoprofen, or dipyrone in pain control after septoplasty and found that a higher opioid requirement was seen in the control group compared with the NSAID group. Despite the use of these opioids, the average visual analogue scale score in the first 8 h was 30 mm or above. Half of the patients in the diclofenac group required opioids, and the incidence of nausea and vomiting was 47.5% and 35% in the placebo group, respectively. Karaman et al. [17] found an average verbal analogue scale score of 4 or above in a control group during the first 12 h after septoplasty. The present study used a combination of tramadol and diclofenac 30 min before the termination of the procedure. This combination has already been reported to be effective in postoperative analgesia [18, 19]. Despite the use of tramadol and diclofenac, the results revealed higher NRS values in the control group compared to the other treatment groups. This is consistent with the findings of previous studies [16, 17]. These findings highlight the importance of pain control in patients undergoing septoplasty in the first hours. The increased incidence of nausea in the control group may be due to the increased pethidine requirement. Nausea occurred frequently within the first postoperative hour shortly after the administration of pethidine. The nausea may be due to the increased administration of ondansetron in the control group.

When pregabalin was added to a multimodal analgesic approach to postoperative pain management, these properties of pregabalin lead to increased analgesic efficacy and decreased opioid consumption [4, 20, 21]. Jokela et al. premedicated patients with 75 mg or 150 mg of pregabalin 1 h before gynaecological laparoscopic surgery and reported better analgesia with a single oral dose of 150 mg of pregabalin in the postoperative period [22]. Mathiesen et al. demonstrated decreased postoperative pain scores with 300 mg of pregabalin in patients undergoing tonsillectomy surgery [4]. In the present study, 300 mg of pregabalin was added to the multimodal analgesic regimen of tramadol plus diclofenac. The addition of pregabalin was associated with lower NRS scores compared to the control group. In addition, the present study showed that opioid consumption and rescue analgesia were reduced in patients receiving pregabalin.

Dexamethasone is a potent synthetic corticosteroid derivative with analgesic, antiemetic, and potent anti-inflammatory activities. The use of dexamethasone in combination with other drugs has been reported to increase the antiemetic or analgesic efficacy and to minimise adverse events when it is used as a single dose in postoperative pain management [23]. Mathiesen et al. [4] used 300 mg of pregabalin and 8 mg of dexamethasone preoperatively in adult tonsillectomy cases and reported a significant decrease in opioid consumption and postoperative pain scores. They also found no difference in the incidence of nausea, vomiting, and consumption of ondansetron. However, the authors reported an increased frequency of dizziness in patients receiving pregabalin or pregabalin plus dexamethasone. Another study investigated the analgesic efficiency of the preoperative administration of 300 mg of pregabalin and/or 8 mg of dexamethasone in hip surgery and found a 50% reduction in postoperative morphine requirements in the patients receiving pregabalin [21]. The addition of dexamethasone did not provide any beneficial effects on pain and opioid requirements. de Sousa Santos et al. [19] reported that tramadol plus dexamethasone reduced pain and rescue analgesic requirements in third-molar surgery. Dursteler et al. [24] used this combination in an experimental study and reported that a synergistic interaction of this combination might be useful in postoperative pain control. In our study, the combination of pregabalin and dexamethasone led to lower postoperative NRS scores compared with the control group. However, this difference was significant only in the first postoperative hour. This data shows that NRS scores are equal but the combined use of pregabalin plus dexamethasone reduces tramadol requirements.

Side effects of pregabalin are well tolerated, and the severity of the side effects is dose dependent. In previous studies, the incidence of dizziness and blurred vision due to pregabalin (600 versus 300 mg) use was shown that 70% versus 60% and 63% versus 50%, respectively [25]. We have found fewer side effects (Table 5). The difference of anesthetic technique, operation, and the populations might be the reason of this difference.

In conclusion, preoperative addition of a single dose pregabalin or pregabalin plus dexamethasone to a multimodal analgesic regimen reduces the consumption of opioids and decreases NRS scores after septoplasty surgery. There is no statistically significant difference between the single dose of pregabalin and the addition of dexamethasone to pregabalin in terms of NRS scores, opioid requirements. We think that single dose 300 mg of pregabalin to a multimodal analgesic regimen provides adequate analgesia for only the first postoperative hours after a septoplasty surgery.

## Figures and Tables

**Table 1:** Demographics and clinical characteristics of the patients.

	Group C (control)	Group P (pregabalin)	Group PD (pregabalin + dexamethasone)	*P* value
Number of patients (*n*)	30	30	30	

Age (yr)	30.9 ± 10.7	28.1 ± 10.0	26.6 ± 7.7	0.217
Gender				0.220
Male	22 (73.3%)	24 (80.0%)	18 (60.0%)	
Female	8 (26.7%)	6 (20.0%)	12 (40.0%)	
Body mass index (kg/m^2^)	24.5 ± 3.6	24.3 ± 3.6	23.5 ± 4.1	0.549
ASA status I/II	24/6	24/6	25/5	0.930
Duration of surgery (min)	44.9 ± 20.4	50.8 ± 23.0	57.2 ± 20.4	0.086
Duration of anaesthesia (min)	54.6 ± 22.2	59.9 ± 23.9	66.4 ± 21.7	0.135
Remifentanil during anaesthesia (ug)	400 (160–1040)	400 (150–880)	380 (120–1000)	0.820

Values are expressed as mean (SD) or *n* (%).

**Table 2:** Consumption of tramadol (mg).

Hours	Group C (control)	Group P (pregabalin)	Group PD (pregabalin + dexamethasone)	*P* value
0-1	20 (0–20)^a,b^	0 (0–20)^a^	0 (0–20)^b^	0.028
1–12	80 (0–220)^a,b^	20 (0–100)^a^	20 (0–100)^b^	<0.001
12–24	40 (0–80)^a,b^	20 (0–100)^a^	0 (0–100)^b^	0.004

Total	120 (0–280)^a,b^	60 (0–160)^a^	40 (0–180)^b^	<0.001

Values are median (interquartile range).

^a^There was a statistically significant difference between Group C and Group P (*P* < 0.05).

^b^There was a statistically significant difference between Group C and Group PD (*P* < 0.05).

**Table 3:** Mean postoperative pain scores (NRS) for each of the indicated times of evaluation (median and interquartile range).

Hours	Group C (control)	Group P (pregabalin)	Group PD (pregabalin + dexamethasone)	*P* value^a^
0	5 (0–8)^b,c^	3 (0–6)^b^	2.5 (0–7)^c^	<0.001
1	2 (0–4)	1 (0–4)	1.5 (0–3)	0.033
2	1.5 (0–5)^b,c^	0 (0–2)^b^	0 (0–3)^c^	0.004
4	1 (0–4)	0 (0–3)	0 (0–3)	0.009
6	1 (0–5)	0 (0–3)	0 (0–2)	0.013
8	1 (0–3)	0 (0–2)	0 (0–2)	0.028
12	1 (0–3)	0 (0–3)	0 (0–3)	0.010
24	0.5 (0–2)	0 (0–2)	0 (0–4)	0.008

^a^Bonferroni adjustment multiple comparisons. *P* value less than 0.00625 was accepted as statistically significant.

^b^There was a statistically significant difference between Group C and Group P (*P* < 0.01).

^c^There was a statistically significant difference between Group C and Group PD (*P* < 0.01).

**Table 4:** Postoperative rescue analgesics and ondansetron use in the groups.

	Group C (control)	Group P (pregabalin)	Group PD (pregabalin + dexamethasone)	*P* value
Number of patients taking meperidine	17 (56.7%)^a,b^	2 (6.7%)^a^	3 (10.0%)^b^	<0.001
Time to the first meperidin dose				0.767
0-1 h	16 (94.1%)	2 (100.0%)	3 (100.0%)	
1–24 h	1 (5.9%)	—	—	
Number of patients taking ondansetron	16 (53.3%)^a^	7 (23.3%)^a^	14 (46.7%)	0.046
Time to the first ondansetron dose				0.410
0-1 h	11 (68.8%)	3 (42.9%)	10 (71.4%)	
1–24 h	5 (31.3%)	4 (57.1%)	4 (28.6%)	

^a^There was a statistically significant difference between Group C and Group P (*P* < 0.05).

^b^There was a statistically significant difference between Group C and Group PD (*P* < 0.001).

**Table 5:** The incidence of observed side effects in the groups.

	Group C (control)	Group P (pregabalin)	Group PD (pregabalin + dexamethasone)	*P* value
Nausea				
0–2 h	12 (40.0%)^a^	3 (10.0%)^a,b^	11 (36.7%)^b^	0.019
0–24 h	16 (53.3%)^a^	7 (23.3%)^a^	14 (46.7%)	0.046
Vomit				
0–2 h	1 (3.3%)	0 (0%)	2 (6.7%)	0.242
0–24 h	4 (13.3%)	4 (13.3%)	5 (16.7%)	0.916
Dizziness				
0–2 h	6 (20.0%)	5 (16.7%)	5 (16.7%)	0.927
0–24 h	8 (26.7%)	6 (20.0%)	11 (36.7%)	0.349
Headache				
0–2 h	5 (16.7%)	2 (6.7%)	4 (13.3%)	0.461
0–24 h	7 (23.3%)	4 (13.3%)	8 (26.7%)	0.420
Blurred vision				
0–2 h	0 (0%)^c^	5 (16.7%)	9 (30.0%)^c^	<0.001
0–24 h	0 (0%)^c^	5 (16.7%)	11 (36.7%)^c^	<0.001

^a^There was a statistically significant difference between Group C and Group P (*P* < 0.05).

^b^There was a statistically significant difference between Group P and Group PD (*P* = 0.017).

^c^There was a statistically significant difference between Group C and Group PD (*P* < 0.01).
